# Supplementary material for: Are carbon emissions trading and green financial instruments synergistic? -Comprehensive quantitative research based on content analysis
Source: PLoS One. 2024 Mar 7;19(3):e0298601. doi: 10.1371/journal.pone.0298601 (PMC10919601; doi:10.1371/journal.pone.0298601)
Supplement: S1 Data — (ZIP) [file pone.0298601.s001.zip › Data/地级市Z_F工作报告.zip!/╘╞─╧/▒ú╔╜╩╨/└┤╫╘╣1⁄2╓┌║┼í╛┬φ┐╦╩2╛▌═°í┐_2018.docx]

各位代表：

现在，我代表市人民政府，向大会报告工作，请予审议，并请市政协委员和列席人员提出意见。

一、2017年工作回顾

一年来，市人民政府深入学习党的十九大精神，以习近平新时代中国特色社会主义思想为指导，全面贯彻落实中央、省委省政府和市委决策部署，全力以赴稳增长、促改革、调结构、惠民生、防风险，主要经济指标增速位居全省前列。实现生产总值678.9亿元，增11%;固定资产投资(不含农户)881.8亿元，增32.1%;一般公共预算收入62.3亿元、支出238亿元，分别增8.4%、11.9%;社会消费品零售总额224.9亿元，增12.4%;城乡常住居民人均可支配收入分别为30164元、10321元，增8.5%和9.5%;金融机构存贷款余额分别为1111.6亿元、691.1亿元，增12.7%和15.4%;节能减排完成省下达任务。除城乡常住居民人均可支配收入、金融机构存款余额3项指标外，市四届人大一次会议确定的年度目标任务均已完成或超额完成。

(一)产业发展成效显著

工业聚集化发展取得突破。保山工贸园区被列为全省重点打造的10个千亿级产业园区之一;“园中园”签订投资项目协议45个，开工11个，投产8个，完成投资22.33亿元，正成为全市工业聚集发展的先行区、试验区、创新区;轻纺、新材料等产业快速发展，轻纺产业园荣获“全国纺织产业转移示范园”称号。腾冲边合区“园中园”规划建设启动。5个省级重点园区同步发展，全市新增入园企业57户，新增规模工业企业34户，实现规模以上工业增加值139.2亿元，增14.1%。民营企业净增3017户，民营经济增加值增12.3%，占全市生产总值的50.8%。

农业规模化发展取得突破。11个万亩规模农业示范区建设积极推进，流转土地12.51万亩，带动农户2.02万户、6.2万人。重点扶持10户农特产品加工企业上市挂牌，完成股改2户，纳入基金支持8户。新型经营主体加快发展，“三品一标”认证企业达76户，新增省级龙头企业10户、市级龙头企业27户、市级示范家庭农(林)场96个、省级农民专业合作示范社20个，8个专业合作社被评为国家级示范社。粮食总产实现14年连增。实现农业增加值161.6亿元，增6.2%。

旅游品牌化发展取得突破。启动高黎贡山“东方黄石公园”项目规划，中心城市“三个万亩”国家生态旅游示范区创建通过省级评定，松山战役旧址公园、玛御谷小镇等项目建设稳步推进，成功举办第5届世界围棋名人争霸战、腾冲国际马拉松、高黎贡山超级越野赛和国际观鸟节，杨善洲精神教育基地入选首批“中国红色地标”，滇西抗战纪念馆等4个景区入选全国红色旅游经典景区名录。“永子”获中国驰名商标，腾冲旅游、龙陵石斛、昌宁红茶入选全国区域品牌价值评价百强榜。腾冲全域旅游全国示范区创建和旅游市场秩序整治成效明显。全市共接待旅游者2511.36万人次，实现旅游总收入263亿元，分别增36.9%、52.3%。

服务业发展取得突破。保山被列为全国电子商务进农村综合示范整体推进市，滇西电子商务产业园成为省级示范基地，全市电子商务交易额达41.77亿元，增48.1%。金融保险、商贸物流等服务业健康发展。实现第三产业增加值274.2亿元，增10.9%。

(二)发展条件不断优化

路网建设加快推进，保泸、昌保等8条高速公路全部开工，杭瑞高速保山坝区段3个立交改造工程顺利启动，提升改造农村公路1004公里，隆阳勐赖大桥即将建成通车，昌宁被列为全国“四好”农村公路示范县和城乡交通运输一体化示范创建县，大瑞铁路建设取得新进展，中心城市有轨电车项目前期工作进展顺利。航空网建设加快推进，保山机场、腾冲机场改扩建步伐加快，腾冲口岸机场建设稳步实施，两个机场新开通省外航线9条，昌宁通用机场开工建设，其他4个通用机场前期工作有序开展。能源网建设加快推进，500千伏永昌输变电工程开工建设，腾冲、施甸、龙陵国家级天然气利用试验示范区支线站场基本建成。水网建设加快推进，启动保山坝、腾北大型灌区前期工作，施甸红谷田等5座中小型水库加快建设，建成腾冲花园水库等9件抗旱水源工程，新增库容1840万立方米，新建五小水利工程2.08万件，受益41.2万人。互联网建设加快推进，3G网络实现全覆盖，4G网络覆盖到重点集镇，村委会全部通光缆，大数据库和政务信息资源交换共享试点获得成功，信息惠民应用初见成效。

(三)城乡环境明显改善

城市生态化取得突破。中心城市“三个万亩”建设成效显著，青华海万亩生态湿地公园被列为国家湿地公园建设试点，东山森林公园“园中园”基本建成，万亩生态观光农业园被列为国家田园综合体建设试点和全省首家国家农业公园创建单位，保山华大智慧农业科技示范园开工建设。昌宁城北湿地、龙陵龙山湖等一批城市公园建成开放。腾冲、板桥国家新型城镇化试点有序推进，潞江镇、和顺镇被列为全国第二批特色小镇，开工建设特色小镇16个，认定全市首批美丽乡村14个，102个传统村落列入美丽宜居乡村建设。申报创建国家级生态乡镇51个、省级生态文明乡镇70个，保山被列为全国第二批生态文明示范工程试点。严格落实污染减排目标责任制，在全省率先推广使用新能源公交车365辆，投放共享单车4000辆。土地节约集约利用水平显著提高，获国务院通报表扬。城镇化率提高到45%。

城乡人居环境整治提升全面展开。“四创两争”卓有成效，保山中心城市成功创建国家卫生城市，被列为全国“城市双修”试点城市、全国首批“公交都市”创建示范城市;腾冲成功创建全国文明城市、国家卫生城市和生态文明城市;昌宁、龙陵成功创建省级生态文明城市，隆阳创建省级生态文明城市通过验收，昌宁获国家园林县城称号。设立市、县、乡、村四级河长1750人，隆阳红花河等3条城市黑臭水体修复工程和施甸、腾冲、昌宁城市河道治理项目全面启动。棚户区改造新开工14073套(户)，货币化补偿12645套(户)，基本建成18112套。开工地下综合管廊19条，建成63.6公里，中心城市地下综合管廊建设工程获中国人居环境范例奖。

(四)脱贫攻坚扎实推进

投入各项扶贫资金150亿元，增9.5%。严格开展贫困对象动态管理，建档立卡贫困人口、易地搬迁对象识别更加精准。建设易地搬迁安置点195个，搬迁入住14320人。开工农村危房改造10300户，竣工7856户。大力发展林果、养殖、中药材等特色产业，切实抓好陡坡地治理和退耕还林，完成营造林23万亩，木本油料提质增效10万亩，实现生态建设、产业发展与脱贫攻坚互促共进。积极推进教育扶贫，贫困家庭学生就学补助、免除学杂费等政策实现全覆盖。大力推进健康扶贫，建档立卡户城乡居民基本医疗保险和大病补充医疗保险参保率、家庭医生签约率均达100%。贫困人口低保、养老保险和贫困特殊人群集中供养做到应保尽保。高黎贡山移民工作稳步开展。直过民族和人口较少民族帮扶工作扎实推进。完成33个贫困村退出、5.9万人脱贫任务。

(五)社会事业稳步发展

投入民生类资金181.8亿元，占一般公共预算支出的76.4%。创建滇西应用技术大学珠宝学院等5个省级创业平台，开发就业岗位3.2万个，城镇新增就业2.77万人，城镇登记失业率控制在3.5%以内。社会保障体系不断健全，城乡居民医疗保险、养老保险法定人群实现全覆盖，城乡低保精准识别和动态管理等救助制度进一步完善，村级养老服务中心和腾冲省级医养结合示范点建设顺利推进。教育“全面改薄”工程深入实施，中小学入学率、巩固率、毕业生升学率均超过全省考核指标，各类教育协调发展。公立医院药品加成全部取消，市人民医院迁建基本完成，市第二人民医院、市妇幼保健院、隆阳区中医院等补短板项目启动前期工作，基层中医药工作走在全省前列，腾冲成功创建基层中医药工作先进市。人口和计划生育政策全面落实。科技水平不断提高，保山工贸园区被确定为国家知识产权试点园区，新增省级高新区1个、可持续发展试验区1个、院士专家工作站5个，新获国家高新技术企业认定10户、省科技型中小企业18户。文化惠民工程扎实开展，基层综合文化服务中心试点建设全面推开，文化对外交流成效显著，广播电视覆盖率达99%，村级农家书屋实现全覆盖。体育事业发展加快，被命名为国家田径单项中长跑和竞走项目奥林匹克高水平后备人才基地，保山运动员首夺全运会冠军，腾冲成为国家体育产业示范基地。认真落实民族宗教和侨务政策，民族团结进步示范点建设扎实推进。基层治理水平不断提高，“6995”“995”和网格化管理取得实效。信访源头治理、积案化解进一步加强。公共法律服务体系逐步完善，全社会尊法学法守法用法意识不断提高。安全生产、食品药品安全责任制严格落实。公安信息化建设应用成效显著，预防和打击犯罪、防范和处理邪教、反恐维稳更加有力，社会更加和谐稳定。国防动员领域改革稳步推进，兵役征集质量逐步提高。妇女儿童、史志、档案、人防、气象、水文、地震、红十字、残疾人等事业健康发展。

(六)改革开放取得突破

重点改革深入推进。深化供给侧结构性改革，化解水泥熟料产能45万吨，房地产去库存45.49万平方米，政府债务得到有效管控，降成本、补短板有序推进。保山成为全省首家全国质量强市示范城市。农村承包土地“三权分置”改革加快实施，确权登记颁证工作稳步开展，产权流转交易市场基本建立。不动产统一登记工作进展顺利。电力体制改革取得突破，实现厂网分开，保山电力整体进入云南电力交易市场，工贸园区配售电公司完成组建，“园中园”获批国家增量配电业务改革试点。市属投融资公司转型升级有序推进，市级经营性国有资产集中统一监管启动实施。“放管服”改革力度加大，市级行政审批项目从2016年的206项精简到116项，“双随机一公开”监管模式全面推行。承担行政职能事业单位改革试点工作通过中央编办验收。商事制度改革成效明显，注册登记实现“多证合一”、全程电子化。预算管理制度等财税领域改革稳步推进。施甸入列全国首批殡葬综合改革试点。

体制机制创新卓有成效。创新投融资机制，实施“51+49”混合所有制股权方式和“10+3”PPP融资方式创新，筹集到位6.48亿元产业发展基金支持重点产业发展，投融资能力逐步提升。创新园区管理机制，5县市区共建“园中园”推动工业聚集化发展路径受到全省关注。创新土地流转机制，流转土地36.81万亩。创新城市管理机制，中心城市数字化管理和综合指挥平台建成试运行，市政基础设施管护市场化稳步推行，综合执法体系和网格化管理模式不断健全。创新开放发展机制，口岸监管实现信息互换、监管互认、执法互助，通关时间压缩1/3，通关便利化水平明显提高。

开放合作水平稳步提升。缪达经济贸易合作区项目通过缅甸国家投资委员会最终审批，“一线两园”开放发展新格局正在形成。组织企业开展保山至曼德勒“产品通关”之行，中方人员持边境通行证的通行范围扩大到密支那。密班公路示范段顺利建成，腾冲航空口岸、木城通道建设有序推进。完成进出口总额3.2亿美元，增21.7%，边民互市贸易货值达9.1亿元，增46.8%。实施“5+8”标准厂房租购、“30+37”电价优惠、“5+5”购房扶持等优惠政策，成功引入一批大型知名企业，签约重点产业招商项目95个，引进市外到位资金800.6亿元，增24.6%。

(七)行政效能全面提升

加快推进地方立法，《保山市昌宁田园城市保护条例》获批实施。主动接受市人大及其常委会的法律监督、工作监督和市政协的民主监督，办理人大代表建议109件、政协委员提案191件，面商率、办复率达100%。深化政府信息公开，健全政府法律顾问和重大决策专家咨询、公示听证等制度，广泛听取各民主党派、工商联、无党派人士、各人民团体和老干部的意见建议，行政决策水平稳步提高。推行“主题+专题”会议、10项重点工作专项评价通报、10项重大项目督查3种办法，狠抓国务院大督查迎检整改和跟踪落实工作，严肃整治不作为、乱作为、慢作为，行政问责干部120名，政纪处分40人。出台提高行政效率9项措施，加快推进政务服务标准化建设，30%的事项实现“最多跑一次”，公共资源交易实行全程电子化，中介超市运行逐步规范，群众对效能政府建设的满意度不断提高。

一年来取得的成绩，是省委省政府和市委坚强领导的结果，是市人大及其常委会、市政协监督支持的结果，是全市广大干部群众奋力拼搏的结果。在此，我代表市人民政府，向全市广大干部群众、省属驻保单位、驻保人民解放军、武警官兵，向关心支持保山发展的社会各界人士，表示衷心的感谢!

我们也清醒地看到，发展不平衡、不充分的问题在保山仍然突出，推动全市跨越发展仍然面临不少困难。一是发展质量不够好，产业结构不够优，在大项目、大企业、大品牌的引进上没有取得实质性突破;二是脱贫攻坚任务艰巨，基本公共服务投入不足，民生事业欠账较多，城乡居民收入差距较大，人居环境有待提升;三是不稳定、不和谐、不安全的因素依然存在，维护社会和谐稳定任务繁重，治理体系有待完善，治理能力有待提升;四是推进改革创新的能力、工作作风和担当精神与新时代新目标的要求还有不小差距。对这些困难和问题，我们一定高度重视，采取有力措施加以克服和解决。

二、2018年工作计划

今年是贯彻落实党的十九大精神的开局之年，是改革开放40周年，是决胜全面建成小康社会、实施“十三五”规划承上启下的关键一年。做好今年的政府工作，必须以习近平新时代中国特色社会主义思想为指导，全面贯彻党的十九大、中央经济工作会议、省委十届四次全会和市委四届四次全会精神，坚持党对政府工作的全面领导，坚持稳中求进工作总基调，坚持新发展理念，紧扣主要矛盾变化，按照高质量发展要求，以供给侧结构性改革为主线，大力弘扬“跨越发展、争创一流，比学赶超、奋勇争先”精神，深入落实省委省政府对保山的发展要求，更加注重发展的开放性、聚集性、持续性、创新性、跨越性，抓重点、补短板、强弱项，推动工业聚集化、农业规模化、城市生态化、旅游品牌化成规模，带动实体经济、脱贫攻坚、基础设施、改革开放、民生保障上水平，奋力开创新时代保山跨越发展新局面。

经济社会发展主要目标建议为：生产总值增10%以上;固定资产投资(不含农户)增20%以上;一般公共预算收入增6%以上;社会消费品零售总额增12%以上;金融机构存贷款余额分别增15%以上;城乡常住居民人均可支配收入分别增10%和12%;引进市外到位资金增20%以上;进出口总额增15%以上;城镇登记失业率控制在4.3%以内;实现9.1万建档立卡贫困人口脱贫;居民消费价格涨幅控制在3%以内;完成省下达的节能减排任务。

围绕上述目标，全力抓好10项重点工作：

(一)全力推进工业聚集化

强力实施“五个一百”。围绕“聚千户企业、创千亿园区”目标，加大招商引资力度，推动符合保山工贸园区及“园中园”产业定位、入园标准的企业入园发展，引进60个以上投资上亿元的签约项目，聚集100户销售收入2000万元以上的企业，将“园中园”打造成全省招商引资示范区。强化融资工作，推动保山工贸园区融资平台获批2A以上信用评级，争取金融机构更大支持，实现融资100亿元。出台企业高端人才引进管理办法，完善人才培引机制和政策措施，畅通社会专业人才引进渠道，引进企业高端人才100名。加快推进园区水、电、路、仓储物流等基础配套设施建设，提升园区综合承载能力，确保规划路网全部连通，主干道和重点项目、重点企业连接道路全面硬化，新建标准厂房100万平方米，完成非电工业投资100亿元。

着力提升园区经济实力。打造“水电硅材工业牌”，依托西安隆基、中科钢研等企业，加快形成以工业硅、多晶硅、单晶硅、太阳能组件等为一体的硅产业链。依托现已形成的纺纱、服装服饰加工、鞋业制造等产业，加快形成轻纺产业集群。推进路华锂离子电池(二期)等在建项目，争取昌宁年产500万套血液透析成套耗材生产基地等项目年内开工，力促施甸富士通科技产业园等一批项目投产达产，确保保山工贸园区签约项目开工50个以上、投产40户以上，实现园区规模以上工业总产值120亿元。加快建设腾冲边合区“园中园”50万平方米标准厂房，推进水长工业园区标准厂房(二期)等项目建设。确保全市规模以上工业增加值增13%以上。

(二)全力推进农业规模化

激发农村发展活力。围绕“产业兴旺、生态宜居、乡风文明、治理有效、生活富裕”要求，科学制定乡村振兴战略规划，扎实推进50个美丽宜居乡村示范项目和万亩生态观光农业园示范区建设。深化农村土地制度和集体产权制度改革，加快“三权分置”改革，完成农村土地承包权、经营权确权登记颁证工作。推进农业供给侧结构性改革，坚持质量兴农、绿色兴农，打造“绿色生态农业牌”，持续开展“三品一标”认证，完善农林科技推广体系，抓好农业投入品、土壤等专项治理，强化农产品质量和安全监管。推进昌宁省级出口茶叶和隆阳省级农产品质量安全示范区建设。完善乡村治理体系，强化乡村规划管理，加强“三农”工作队伍建设，推进农村生活环境、生产环境、社会环境等综合治理，营造文明健康、积极向上的乡风民风家风。

加快推进种养加销一体化。主攻种养、加工、销售规模化，高质量建设11个万亩规模农业示范区，有序推进构树、优质商品蔬菜生产基地建设。健全土地流转机制，推行专业大户、农民专业合作社、家庭农场、龙头企业带动模式，积极探索宅基地集约开发、农村土地银行、土地股份合作社、田保姆经营模式，提高流转土地使用效益和农民收益。充分发挥产业基金引导作用，完善农产品加工业发展扶持政策，加大企业培育和招引力度，大力推进石斛、核桃、茶叶、咖啡等农特产品精深加工，继续扶持农产品加工企业上市挂牌。健全农业社会化服务体系，带动多层次的适度规模经营，培育农业产业化省级重点龙头企业5户、专业合作社100个。实施农业标准化战略，加快农产品交易市场和农贸市场建设。落实粮食安全行政首长责任制，完成310万亩粮食生产功能区和重要农产品生产保护区划定工作。巩固提升两烟、畜牧等传统产业，加快发展中药材、柑橘等产业。积极发展乡村旅游、商贸物流等农村服务业，促进农村一二三产业融合发展。确保农业增加值增6%。

(三)全力推进城市生态化

全面实施“城市双修”工程。着力打造“生态城市园林牌”，加快推进中心城市“三个万亩”生态廊道工程，确保青华海东湖公园2018年春节前基本建成并向市民开放，东山省级森林公园创建全面启动，万亩现代农业观光园年内开园运营;推进中心城市青阳公园和施甸太阳湖、腾冲小西湖、龙陵龙山湖二期、昌宁茶韵公园等生态公园建设，启动施甸、昌宁城市公园“园中园”建设。统筹推进“四创两争”，加强城市数字化、网格化、精细化管理，巩固创文、创卫成果，抓好隆阳、施甸、龙陵省级文明城市和昌宁全国文明城市创建工作，加快腾冲国家森林城市创建，启动龙陵、昌宁省级森林城市和昌宁城北省级湿地公园创建，推进施甸、龙陵、昌宁国家卫生县城创建。启动南苑商场、如意巷等片区城市修补试点项目，完成中心城市道路改造提升，加强城市道路交通管理，扎实创建全国“公交都市”示范城市。加快建设城市综合管廊，每个县(市、区)开工建设1个海绵城市建设示范项目。完成城市生态化项目建设投资40亿元。

着力打造人居环境示范区。以保山坝区为重点，加大违法建筑拆除力度，加强棚改安置小区管理，持续抓好永昌路两侧城市建筑物外立面综合整治提升，启动东河和大保高速保山坝区段、青阳路全线、永昌路和保岫路延长线两侧50米范围内的环境综合整治提升，统筹推进棚户区改造、“厕所革命”、城市面山治理等工作，完成中心城市红花河、大小桥河、西大沟黑臭水体修复和城北防洪河治理。整治提升15条干线公路及108公里旅游公路周边城乡人居环境。抓好施甸河、腾冲大盈江河、龙陵东河、昌宁右甸河等河道综合治理。加快推进腾冲、板桥国家级和3个市级新型城镇化综合试点，高质量建设和顺古镇等16个特色小镇。全力打造全省坝区人居环境整治提升示范区。

加强生态建设和环境保护。大力推进生态文明市创建，确保通过省级认定，启动国家级示范市创建。编制自然资源资产负债表，科学划定生态保护红线，严格耕地保护，加强自然保护区、重要湿地和生态脆弱区域生态保护。启动国土绿化行动，实施退耕还林还草17.5万亩，停止728万亩天然林商品性采伐，推进468万亩公益林保护。全面落实河长制，抓好大气、水、土壤等综合治理。实行最严格的生态环境保护制度，坚决防止和严厉惩处破坏生态环境行为，全力打好污染防治攻坚战，促进优质生态环境共建共享。

(四)全力推进旅游品牌化

打造“健康生活目的地牌”。全面落实高黎贡山“东方黄石公园”建设总体规划，着力构建“一圈一环一线”旅游大发展格局。积极整合全市各类自然保护区资源，完成高黎贡山公园规划，按5A级景区标准推进百花岭、林家铺游客中心建设，将青华海创建为国家生态旅游示范区、4A级景区，推进天堂山生态旅游景区等建设项目，着力构建以高黎贡山国家级自然保护区为核心、辐射周边的自然保护区生态旅游圈。抓好高黎贡山摄影小镇等项目建设，着力构建以高黎贡山旅游度假区和怒江大峡谷景区为核心、二级公路为纽带、多点支撑的高黎贡山旅游环。推进善洲小镇、松山小镇、和顺古镇等建设项目，抓好怒江抗战江防遗址保护利用工程，完成施甸七零七至龙陵龙山卡红色旅游公路保护提升工程，扎实开展腾冲和顺5A级景区和滇西抗战纪念馆及国殇墓园、善洲林场4A级景区创建工作，着力构建以弘扬抗战文化、传承艾思奇理论创新精神和杨善洲精神为核心的红色经典旅游线。

深入实施“旅游+”行动。推动旅游与城市生态化融合发展，积极打造城市景区景点和旅游环线，促进城市宜居宜业与宜游同步提升，确保腾冲全域旅游示范区创建通过省级验收。推动旅游与农业融合发展，每个万亩规模农业示范区启动1个休闲观光景点建设。推动旅游与交通融合发展，县(市、区)、高黎贡山旅游度假区分别精心打造1条旅游景观通道。推动旅游与文化、体育及大健康等产业融合发展，抓好腾冲东山中医药健康旅居小镇建设，办好高黎贡山国际观鸟节和超级越野赛、腾冲国际马拉松等国际赛事。推动旅游与信息化融合发展，建设好“一部手机游云南”保山版。持续整顿旅游市场秩序，坚决防治旅游乱象。全年接待旅游者和旅游总收入分别增15%、25%以上。

(五)全力推进实体经济发展

实施企业成长四项行动。实施企业达规行动，全面落实民营经济发展“双10条”，建立市场准入负面清单，出台本土企业发展扶持政策，支持企业做强做大，新增规模以上工业企业30户以上、商贸企业25户以上。实施质量提升行动，弘扬企业家精神、劳模精神和工匠精神，以提升产品和服务质量为主攻方向，加强质量基础设施建设，抓好政府质量奖评选，巩固提升腾冲旅游、龙陵石斛、昌宁红茶、“永子”等知名品牌的市场竞争力，支持企业增品种、提品质、创品牌，新增全国知名品牌创建示范区2个。实施减税降费行动，全面落实减税政策和企业发展扶持政策，降低制度性交易成本，确保企业总体税费负担只减不增。实施服务提升行动，完善挂钩服务规模企业机制，及时帮助企业解决生产经营困难，着力构建“亲”“清”新型政商关系，营造公平的市场环境、公正的法治环境，切实维护企业合法权益。

培育产业发展新动能。促进互联网、大数据与实体经济深度融合，加快改造传统产业，积极培育共享经济、数字经济、生物经济等领域的新动能，推动产业向开放型、创新型、绿色化、高端化、信息化转型升级。做强能源工业，推动硅电、铝电联动发展，着力打造云南硅电一体化示范区。合理布局特色小镇主导产业，突出自身特色，积极培育新的增长点。加快发展现代服务业，推进保山工贸园区、腾冲中和片区、猴桥口岸等重点物流园区建设，完善城乡物流配送和快递物流体系，积极推进乡村新型商业中心建设。有序发展汽车、建材等专业市场。强化市场监管和消费维权。积极推进电子商务进农村，实现乡镇电子商务服务站全覆盖。确保第三产业增加值增8.5%以上。

防范化解重大风险。加快建立重大风险防范化解机制，着力推动金融监管、财政、国资、公安、工商等部门形成风险监管整体合力，坚决守住不发生系统性风险的底线。着力防范化解金融风险，落实属地责任，支持市内金融机构化解不良贷款和盘活存量，提升服务实体经济能力，加强互联网金融、民间借贷等薄弱环节监管，坚决打击违法违规金融活动。着力防范化解政府债务风险，全口径管控政府债务，严禁违法违规举债和担保，严禁违法违规购买服务，严禁违规建设楼堂馆所，严格控制增量债务，积极争取中央转移支付和省级资金支持，逐步消化存量债务。着力防范化解国有企业债务风险，强化负债率和资本金约束，推进市场化法治化债转股，盘活企业存量资产，推动投融资平台公司市场化转型、实体化经营，提高经营效益，提升偿债能力。

(六)全力推进脱贫攻坚

全面推进精准脱贫。以脱贫攻坚统揽经济社会发展全局，紧扣“两不愁三保障”目标，聚焦深度贫困地区精准发力，深入推进动态管理、脱贫措施、资金安排、项目实施、责任人员精准到村到户，确保工作扎实、措施落实、成效真实。坚持定性与定量相结合，全力提高脱贫质量，严格识贫脱贫分级审核程序，建立公开公示机制，坚决防止数字脱贫、弄虚作假，确保识贫脱贫得到群众认可、经得起各方检验，群众满意度达到90%以上。加大扶志力度，激活脱贫内生动力，增强社会扶贫、企业帮扶等各方合力。统筹非贫困村、非建档立卡贫困户的扶持，抓好直过民族脱贫攻坚行动和人口较少民族帮扶工作。扎实开展脱贫攻坚作风建设年活动，严格执行评价通报制度，推动各方责任落地落细。深入开展扶贫领域专项治理，加强扶贫资金跟踪审计，严肃查处贪占挪用扶贫资金行为。高质量完成90个贫困村退出、9.1万建档立卡贫困人口脱贫任务，实现昌宁整县脱贫摘帽，坚决打好新时代第一场硬战，确保进入全省前列。

统筹推进“五个一批”。加大产业扶贫力度，强化规模农业带动作用，密切新型经营主体与贫困群众的利益联结，有效解决贫困群众就业问题，拓展外出务工、乡村旅游、电商扶贫等增收渠道，确保产业到户全覆盖、贫困群众有长期稳定收入。扎实推进易地扶贫，严格标准、严控成本、严守红线，搬迁建档立卡贫困人口34666人，完成“四类重点对象”危房改造8000户，加快实施国开行、浦发行项目，确保脱贫退出村基础设施全面达标。抓好生态扶贫，大力发展林果产业和林下经济，生态建设项目向贫困村倾斜，让更多贫困群众参与生态管护获得稳定收入。着力推进教育扶贫，坚持扶贫与扶智相结合，积极开展就业技能培训，落实贫困家庭学生就学减免资助政策，杜绝因学致贫、因贫辍学。大力实施健康扶贫，切实降低贫困群众就医负担。充分发挥农村低保的兜底保障作用，落实养老、残疾人等扶贫政策措施，实现贫困户社会保障政策应享尽享。

(七)全力推进基础设施建设

加快建设内畅外通的路网。高效推进8条高速公路建设，完成杭瑞高速保山坝区段3个立交改造工程，抓好国省道和“四好”农村公路建设，新改建农村公路1300公里，完成交通建设投资100亿元。协调推进大瑞铁路保山境内段建设，加快推进芒市至腾冲猴桥铁路前期工作，确保中心城市有轨电车项目上半年开工。

加快建设高效便捷的航空网。抓好保山机场改扩建、腾冲机场二期改扩建及口岸机场建设。加快推进昌宁通用机场建设和其他4个通用机场前期工作。争取新开通航线5条以上，累计达到27条以上。完成机场建设投资1.8亿元。

加快建设优质安全的能源保障网。抓好500千伏永昌输变电工程、220千伏骨干电网和110千伏以下配电网规划建设，推进农网城网改造项目，提高供电保障能力。抓好6个中小型水电站建设，新增电力装机28.24万千瓦。积极应对电力市场变化，着力降低购电成本。加快推进天然气利用试验示范区建设，确保天然气用气量达到600万立方米。完成能源建设投资10亿元。

加快建设安全可靠的水网。编制中心城市和保山工贸园区用水保障规划，整合城区供水，积极推进“引澜入保”等一批水利工程。加快施甸红谷田等3座中型水库和13座小(一)型水库建设，推进隆阳阿贡田等4座中型水库和16座小(一)型水库项目前期工作，确保龙陵段家坝等3座中型水库和6座小(一)型水库建成发挥效益。新开工一批农田水利、农村饮水工程，巩固提升39万农村人口饮水安全。完成水利建设投资26.2亿元。

加快建设共享高效的互联网。推进省电子政务保山灾备中心和“保山云数据中心”建设，加快“三网融合”试点步伐。建成智慧交通信息化服务平台，推广信息惠民应用。新建通信基站650个，光纤接入能力覆盖城区和所有建制村。支持5G网络布局和发展，确保农村4G网络覆盖面达到90%以上，主要景点免费WIFI实现全覆盖。完成信息基础设施建设投资4亿元。

(八)全力推进改革开放

深入推进改革创新。抓好国资国企、电力体制、商事制度等经济领域改革，深化“放管服”改革，统筹推进事业单位、生态环保、社会事业、统计管理、殡葬等领域改革。深化投融资机制创新，扩大市属投融资公司资产规模，确保市级经营性国有资产集中统一监管取得阶段性成果;充分发挥“51+49”混合所有制股权方式和“10+3”PPP融资方式的综合效应，争取更多项目列入国家和省级项目试点;全面落实“1+5”共建共享发展机制，有效解决重大项目落地难问题。深化园区管理机制创新，组建工业聚集化发展专家委员会，成立保山工贸园区集中审批服务中心，健全重大招商项目全程代办和联审联批制，提高工业聚集发展服务水平。加大研发投入力度，大力培育科技创新主体，加快建立以企业为主体、市场为导向、产学研用深度融合的技术创新体系，新申报认定高新技术企业10户以上。

扩大对内对外开放。主动服务和融入“一带一路”，积极推进面向南亚东南亚国际大通道境内段建设，抓好曼德勒缪达保山工业园区服务机构组建工作，完成12万平方米标准厂房及相关配套设施建设，引进5户以上有实力的企业落地，推进缅甸密支那经济开发区和中缅猴桥—甘拜地跨境经济合作区建设，着力打造“两线两园开放牌”。继续组织“产品通关”之行，提高通关便利化水平。深化与周边国家经贸、文化、教育等领域的交流合作，扩大边民互市规模。强化与长江经济带、珠三角和环渤海等重点区域的交流合作，围绕支柱产业定向定位定点精准招商，着力提高开放发展水平。

(九)全力推进民生保障

优先发展教育事业。促进义务教育公平均衡，完成“全面改薄”任务，深入推进素质教育。做好普及高中教育规划并着力实施，推进普通高中扩容提质。大力发展学前教育，不断提高保教能力，增强学前教育的公益性和普惠性。积极发展高等教育、职业教育，启动保山学院理工学院和国家华文教育基地建设，办好滇西应用技术大学珠宝学院，推进产教融合、校企合作。统筹抓好民族教育、特殊教育、继续教育和老年教育，支持和规范社会力量办学，促进民办教育健康发展。加强师德师风建设，培育高素质教师队伍，倡导全社会尊师重教。

加强就业和社会保障。加大创业政策扶持，支持高校毕业生返乡创业，开发就业岗位2.9万个，新增城镇就业2.4万人。抓好隆阳区农民工返乡创业试点。推进全民参保计划，基本养老保险和医疗保险参保率稳定在95%以上。统筹城乡社会救助体系，完善最低生活保障、特困人员供养、灾害救助、医疗救助、临时救济制度。健全养老服务体系，推进养老机构改革，抓好医养结合试点。加快建立多主体供应、多渠道保障、租售并举的住房制度，有效满足不同层次的住房需求。实施妇女儿童关爱行动，建成市级残疾人康复服务中心，积极发展志愿服务和慈善事业。

提升医疗卫生服务。深化医药卫生体制改革，健全药品供应保障制度。启动中医专直属附属医院建设项目，推动市人民医院与高水平医疗机构合作办医，推进县级中心医院提质达标。完善城乡医疗卫生服务体系，抓好重点学科和专家工作站建设，加快发展医联体，推进分级诊疗和家庭医生签约服务，促进医疗资源向基层下沉。加强卫生计生监督执法，规范医疗服务市场秩序。加强公共卫生和疾病预防控制，深入开展爱国卫生运动。积极发展中医药事业，争创全国基层中医药工作先进市。全面落实人口和计划生育政策，提高妇幼保健水平。

繁荣文化体育事业。推动社会主义文化繁荣兴盛，弘扬中华优秀传统文化，培育和践行社会主义核心价值观。完善公共文化基础设施，抓好保山文化艺术中心建设和国家公共文化服务体系示范区复评巩固工作。打造一批地方文化、民族文化精品，促进文化交流。支持文化产业发展，加大文物和非物质文化遗产保护力度。广泛开展群众性文体活动，倡导全民阅读，净化网络空间，持续开展七彩云南全民健身工程，提高各类体育场馆和公共健身设施开放利用水平。

强化社会治理创新。深入开展“七五”普法，加快建立完备的公共法律服务体系。全面宣传贯彻执行新修订的省信访条例，认真负责处理好人民群众来信来访，积极化解信访积案。大力推动综治中心建设，抓好矛盾纠纷预防、化解和处置工作。强化安全生产责任制，深化安全生产领域改革，提升防灾减灾救灾能力。加强食品药品安全监管，保障人民群众饮食用药安全。实施好民族团结进步示范建设项目，推进沿边三年行动计划，依法管理民族宗教事务，确保民族团结、宗教和睦、边疆稳定。加强平安保山建设，深入开展平安创建活动，启动“阳光片区”建设，完善社区网格化管理机制，推进公安信息化建设应用“三个一”工程，打好禁毒防艾、反恐、反邪教人民战争，扎实开展扫黑除恶专项斗争，强化边境整治，严厉打击非法集资、网络诈骗和黄赌毒黑拐骗等违法犯罪行为，持续提高人民群众安全感和满意度。加强兵役、双拥、人防、退伍军人安置等工作，推进军民融合发展。做好史志、侨务、气象等工作，支持工会、共青团、妇联、科协、社科联等群团组织改革发展。

(十)全力推进政府自身建设

坚持党的全面领导。树牢“四个意识”，坚定“四个自信”，坚决维护党中央权威和集中统一领导，坚决维护习近平总书记在党中央和全党的核心地位，思想上认同核心、政治上维护核心、行动上紧跟核心。坚决服从市委领导，始终与市委保持高度一致，做到市委有号召、政府有行动，市委有安排、政府有落实，有令必行、有禁必止，确保党中央国务院、省委省政府和市委各项决策部署执行坚决、落实有效。

全面增强执行本领。以深入学习宣传贯彻落实党的十九大精神为主线，扎实开展“不忘初心、牢记使命”主题教育，用习近平新时代中国特色社会主义思想武装头脑、指导实践、推动工作。坚持问题导向，紧扣全市跨越发展最核心、最关键、最紧迫的重大问题，围绕群众最关心、最直接、最现实的利益问题，加强调查研究，强化教育培训，着力培养金融、工业、信息化等专业能力，全面提升改革创新、群众工作、驾驭风险等“八种本领”，以崭新的精神状态和奋斗姿态推动保山跨越发展。

全力提升行政效能。以“行政效能提升年”为主题，着力整治漂、虚、懒、散、粗、怯、奢现象，打通阻碍落实的堵点，立起高效落实的规矩，确保工作在状态、落实见效率。坚持“主题+专题”会议、重点工作评价通报和重大项目督查制度，比出精神、比出效率、比出成绩，进一步激发县市区五马竞争、争创一流的强大动力，加快形成各部门千帆竞发、奋勇争先的生动局面。坚持言必信、行必果，发扬钉钉子精神，推行工作任务清单制、销号制管理，改进督查督办，强化跟踪问效，确保事事有回音、件件有落实。加快推进“互联网+政务服务+公共资源交易”，健全线上线下协同高效服务体系，深入开展减证便民行动，力争60%的事项让办事群众“最多跑一次”。

加快建设法治政府。扎实推进地方立法，积极做好《保山市龙陵松山战役旧址保护条例(草案)》《保山市城市环境卫生管理条例(草案)》的调研起草工作。主动接受市人大及其常委会的法律监督、工作监督和市政协的民主监督，抓实重大事项人大审议决定和规范性文件备案审查，办理好人大代表议案建议和政协委员提案。落实重大行政决策终身责任追究制，严守合法性审查、社会公示听证等法定决策程序，健全政府法律顾问和专家咨询制度，扎实开展协商民主，广泛听取各方意见，推进决策科学化、民主化、法治化。认真履行法治政府建设职责规定，坚持法定职责必须为、法无授权不可为，严格执行权责清单，带头尊法学法守法用法，严格落实行政执法责任追究制，推进综合执法、规范公正文明执法，着力提高依法行政水平。推进政务公开规范化标准化，自觉接受社会、司法、舆论等各方监督，让权力运行更加阳光透明。

深入推进党风廉政建设。以永远在路上的韧劲和执着，把全面从严治党要求不折不扣落实到政府工作的全过程全领域。认真贯彻中央八项规定精神，严格执行省市实施办法，着力整治形式主义、官僚主义等“四风”问题新表现新动向，持之以恒正风肃纪。严格预算管理，严控“三公”经费，勤俭节约办事。恪守“短、实、新”要求，切实改进文风会风。推动容错纠错机制落地，为干事者鼓劲、为担当者撑腰。全面落实管党治党“两个责任”，始终把政治建设摆在首位，以政治纪律带动其他党纪政纪全面严起来。抓好监察体制改革，实现政府公职人员监察全覆盖。强化脱贫攻坚、征地拆迁、工程建设、惠民政策等重点领域监管，加强审计监督，严肃执纪问责，深入推进反腐败斗争，严肃处理各种违法违规行为，确保政府廉政、干部廉洁。

各位代表!进入新时代，保山应有新气象;开启新征程，政府须有新作为。让我们更加紧密地团结在以习近平同志为核心的党中央周围，在省委、省政府和市委的坚强领导下，不忘初心、牢记使命，勇于开拓、锐意进取，奋力开创新时代保山跨越发展新局面，为决战脱贫攻坚、决胜全面建成小康社会而不懈奋斗!
